# Painful nodules on the lower legs

**DOI:** 10.1016/j.jdcr.2024.06.032

**Published:** 2024-07-08

**Authors:** Chiamaka Ohanenye, Nicholas D. Brownstone, Sylvia Hsu

**Affiliations:** Department of Dermatology, Temple University Hospital, Philadelphia, Pennsylvania

**Keywords:** pancreatic panniculitis, pancreatitis

## History

**Fig 1:**
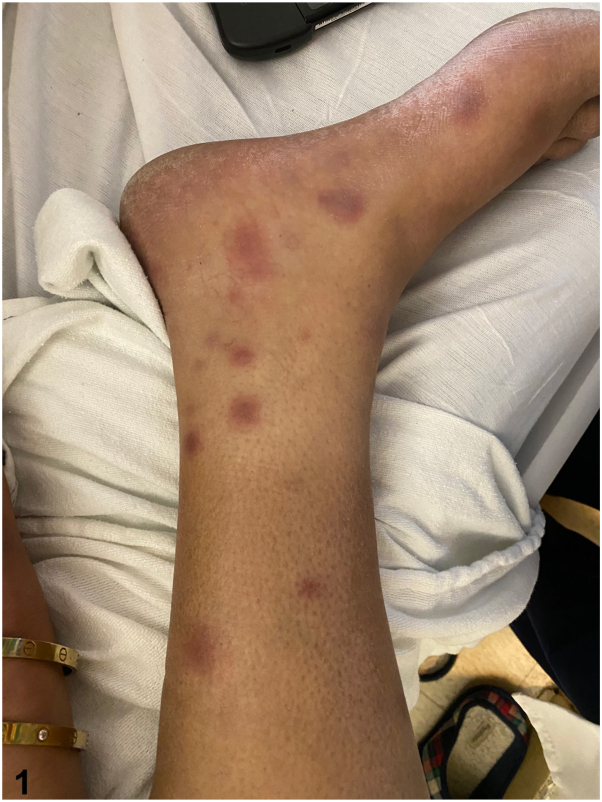

**Fig 2:**
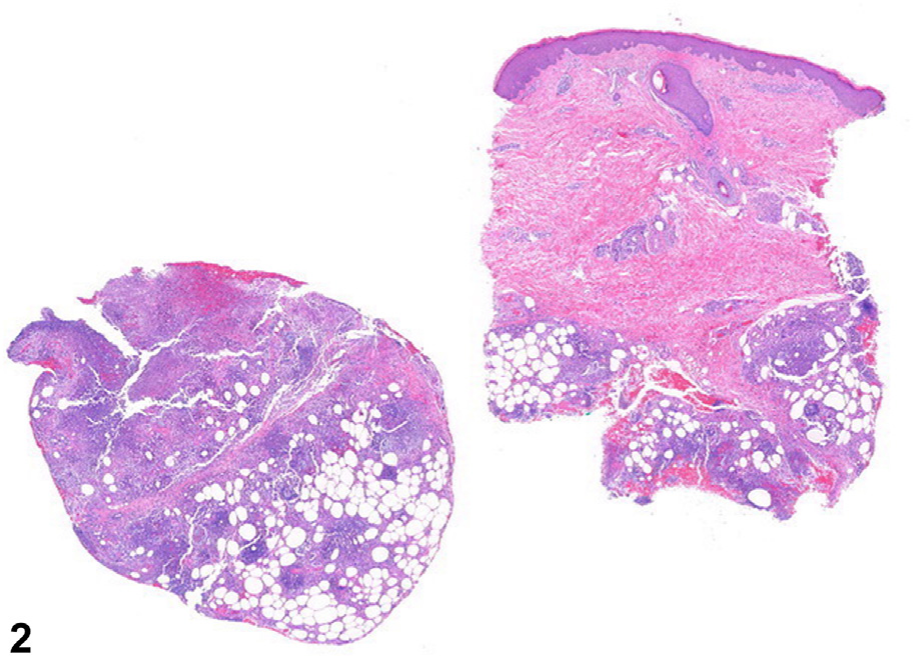

A 44-year-old man with no prior medical history presented with 2 days of dyspnea and painful, non-pruritic nodules on the bilateral pretibial areas and dorsal feet. The patient was tachypneic and hypoxic and was admitted to the hospital. Laboratory studies were notable for amylase and lipase levels of >1302 U/L (ref range, 1–105 U/L) and 10,287 U/L (ref range, 73-393 U/L), respectively. Computed tomography scans showed a large right-sided pleural effusion and an epigastric fluid collection. Physical exam showed tender, erythematous, subcutaneous nodules without ulceration on both shins and dorsal feet (Fig 1), and histological findings are shown (Fig 2).

**Question 1: What is the most likely diagnosis?**

A. Alpha-1 antitrypsin deficiency panniculitis
B. Lupus panniculitis
C. Pancreatic panniculitis
D. Poststeroid panniculitis
E. Erythema nodosum

**Answers:**

A. Alpha-1 antitrypsin deficiency panniculitis – Incorrect. This is a manifestation of alpha-1 antitrypsin deficiency, which is an autosomal codominant condition that primarily affects the lungs and liver. Histologically, alpha-1 antitrypsin deficiency panniculitis is characterized by liquefactive necrosis of the dermis and subcutaneous septa, which is inconsistent with the histology in this case.^1^
B. Lupus panniculitis – Incorrect. Lupus panniculitis is a form of chronic cutaneous lupus erythematosus. Ten percent of lupus panniculitis patients have signs of systemic lupus erythematosus. The histology is incorrect for lupus panniculitis. Histologic findings of lupus panniculitis are characterized by hyaline necrosis and lymphoplasmacytic infiltrates.^1^
C. Pancreatic panniculitis – Correct. Pancreatic panniculitis is a rare cutaneous manifestation of pancreatic disease characterized by painful, erythematous, subcutaneous nodules that can ulcerate and produce an oily substance. In this case, the patient had imaging findings and labs consistent with acute pancreatitis.^1^
D. Poststeroid panniculitis – Incorrect. This occurs within 10 days of rapid tapering or withdrawal of systemic corticosteroid therapy,^1^ neither of which the patient experienced.
E. Erythema nodosum – Incorrect. This is one of the most common forms of panniculitis, most often idiopathic in nature, but can also be caused by sarcoidosis, inflammatory bowel disease, drugs, and infectious etiologies, such as streptococcal infection.^1^ The histology is incorrect for erythema nodosum, which is characterized by septal panniculitis.^1^

**Question 2: Which lab value is most likely to be abnormal in this condition?**

A. Tryptase
B. Antinuclear antibodies
C. Amylase
D. Antineutrophil cytoplasmic antibodies
E. Lipase

**Answers:**

A. Tryptase – Incorrect. Tryptase is an enzyme involved in the degranulation of mast cells, which occurs in anaphylaxis, mastocytosis, and hematological malignancies.
B. Antinuclear antibodies – Incorrect. These antibodies may be elevated in a variety of autoimmune disorders, such as systemic lupus erythematosus, scleroderma, and dermatomyositis.
C. Amylase – Incorrect. Although amylase is an enzyme that is commonly elevated in cases of pancreatic panniculitis, many case reports have shown cases with normal amylase levels.^2^ Evidence shows that elevated amylase is not sufficient for the development of severe pancreatic panniculitis.^3^
D. Antineutrophil cytoplasmic antibodies – Incorrect. This antibody is positive in various vasculitides, such as granulomatosis with polyangiitis, eosinophilic granulomatosis with polyangiitis, and microscopic polyangiitis.
E. Lipase – Correct. Pancreatic panniculitis is hypothesized to be caused by saponification leading to fat necrosis and inflammation, which is believed to be primarily mediated by lipase.^3^ Lipase is most often elevated in cases of pancreatic panniculitis, while other enzymes, such as amylase, phosphorylase, and trypsin, do play a role but are not as consistently elevated.^4^

**Question 3: What histologic finding is pathognomonic for this condition?**

A. Ghost cells
B. Pautrier microabscesses
C. Fibrinoid necrosis
D. Needle-shaped clefts in lipocytes
E. Elongation of rete ridges

**Answers:**

A. Ghost cells – Correct. These have been described as anucleate blue-gray amorphous material surrounded by numerous neutrophils and are commonly seen in pancreatic panniculitis.
B. Pautrier microabscesses – Incorrect. This is an intradermal nest of atypical lymphocytes, characteristic of cutaneous T-cell lymphomas, such as mycosis fungoides.
C. Fibrinoid necrosis – Incorrect. This is described as fibrin deposition within and around the walls of a vessel and is commonly seen in vasculitis, such as leukocytoclastic vasculitis.
D. Needle-shaped clefts in lipocytes – Incorrect. This is a finding seen in sclerema neonatorum and poststeroid panniculitis.
E. Elongation of rete ridges – Incorrect. This is a finding of psoriasis.

## Conflicts of interest

None disclosed.
